# Structure-function-relationship of the APP-E1-domain

**DOI:** 10.1186/1750-1326-8-S1-P59

**Published:** 2013-10-04

**Authors:** Sandra Hoefgen, Sven O Dahms, Dirk Roeser, Manuel E Than

**Affiliations:** 1 Leibniz Institute for Age Research – Fritz Lipmann Institute (FLI), Jena, Germany

## Background

Alzheimer’s disease is one of the most frequent forms of dementia in the elderly population affecting about 25 % of people in the age of 80 to 90 years [1]. Due to the more and more ageing society the importance of dementia is increasing. The brain of affected patients is characterized by the deposition of senile plaques containing the neurotoxic peptide Ab_40-42_ that is derived out of its precursor, the Amyloid Precursor Protein (APP) [2]. Beside its role in Alzheimer’s pathology many physiological functions, like stimulation of synaptogenesis and signal transduction in a receptor-like manner are discussed for APP [3]. However, until now it was not possible to correlate the known structures of subdomains with most of the proposed physiological functions of APP.

## Materials and methods

Using X-ray crystallography we solved the structure of the APP-E1-domain [4] and together with biochemical methods like isothermal titration calorimetry (ITC), analytical gelfiltration, limited proteolysis, static and dynamic light scattering we analysed the dimerization of APP-E1 and two mutated versions of it.

## Results

Here we present the structural and biochemical characterization of the APP-E1-domain showing that the growth-factor-like domain (GFLD) and the copper-binding domain (CuBD) form one closed structural and hence functional entity. We could show concentration dependent self-dimerization of the E1 domain at high and the heparin dependent dimerization at low protein concentrations and set up a model of dimerization, which was also based on crystallographic data. We additionally could prove this model using our E1 mutants.

## Conclusions

We could nicely show the dimerization of the APP-E1 domain and identified the previously described loop region of the growth factor like domain [5] to be essential for this dimerization. Together with our structural data we could for the first time link structure and function of APP.
